# A Mendelian randomization study of the effect of type-2 diabetes on coronary heart disease

**DOI:** 10.1038/ncomms8060

**Published:** 2015-05-28

**Authors:** Omar S. Ahmad, John A. Morris, Muhammad Mujammami, Vincenzo Forgetta, Aaron Leong, Rui Li, Maxime Turgeon, Celia M.T. Greenwood, George Thanassoulis, James B. Meigs, Robert Sladek, J. Brent Richards

**Affiliations:** 1 Centre for Clinical Epidemiology, Lady Davis Institute for Medical Research, Jewish General Hospital, McGill University, Montreal, Quebec H3A 0G4, Canada; 2 Department of Medicine, McGill University, Montreal, Quebec H3A 0G4, Canada; 3 Department of Human Genetics, McGill University, Montréal, Québec H3A 0G4, Canada; 4 Division of General Internal Medicine, Massachusetts General Hospital and Department of Medicine, Harvard Medical School, Boston, Massachusetts 02115, USA; 5 Department of Oncology, Epidemiology, Biostatistics and Occupational Health, and Human Genetics, McGill University, Montreal, Quebec H3A 0G4, Canada; 6 Department of Twin Research and Genetic Epidemiology, King's College London, London SE1 7EH, UK

## Abstract

In observational studies, type-2 diabetes (T2D) is associated with an increased risk of coronary heart disease (CHD), yet interventional trials have shown no clear effect of glucose-lowering on CHD. Confounding may have therefore influenced these observational estimates. Here we use Mendelian randomization to obtain unconfounded estimates of the influence of T2D and fasting glucose (FG) on CHD risk. Using multiple genetic variants associated with T2D and FG, we find that risk of T2D increases CHD risk (odds ratio (OR)=1.11 (1.05–1.17), per unit increase in odds of T2D, *P*=8.8 × 10^−5^; using data from 34,840/114,981 T2D cases/controls and 63,746/130,681 CHD cases/controls). FG in non-diabetic individuals tends to increase CHD risk (OR=1.15 (1.00–1.32), per mmol·per l, *P*=0.05; 133,010 non-diabetic individuals and 63,746/130,681 CHD cases/controls). These findings provide evidence supporting a causal relationship between T2D and CHD and suggest that long-term trials may be required to discern the effects of T2D therapies on CHD risk.

## Abstract (web summary)

In order to effectively design interventions, it is useful to understand the complex interplay between multiple syndromes. Here, Ahmad *et al*. use genome-wide association study data and Mendelian randomisation to examine the influence of Type 2 diabetes and fasting glucose levels on coronary heart disease.

## Graphical abstract

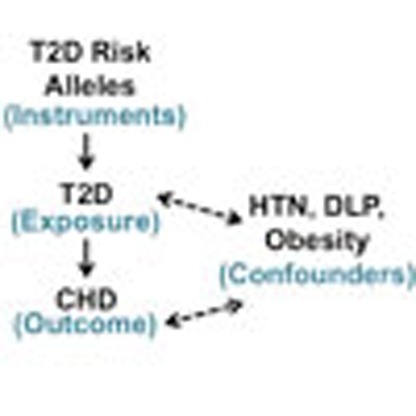

Understanding the role of type-2 diabetes (T2D) in the pathogenesis of coronary heart disease (CHD) is a fundamental problem for the design of effective approaches for preventing cardiovascular disease^1^. T2D is associated with an increased risk of CHD by—two to fourfold in observational studies^2^,^3^, and this effect is independent of known T2D-associated risk factors, such as low-density lipoprotein (LDL) cholesterol, smoking, blood pressure, body mass index (BMI) and inflammatory markers. In non-diabetic patients, the two key metabolic traits associated with T2D, elevated fasting glucose (FG) levels and insulin resistance, are also associated with an increased risk of cardiovascular disease^2^,^4^.

Despite this observational evidence, recent large-scale randomized controlled trials (RCTs) have shown conflicting and inconclusive results on the effect of intensive glucose-lowering therapies on the short-term prevention of CHD in patients with T2D. Four large-scale RCTs^5^,^6^,^7^,^8^ have shown no benefit to intensive glucose-lowering therapy; indeed, one was stopped prematurely because of increased mortality in the treatment arm^5^. Nevertheless, a recent meta-analysis of RCTs has indicated a modest benefit to of glucose lowering on CVD outcomes^9^. Furthermore, a recent follow-up observational study of patients who had been enroled in the ACCORD RCT showed some evidence of benefit of intensive glucose-lowering therapy on cardiovascular outcomes^10^. Finally, leading clinical treatment guidelines for T2D recommend control of blood glucose for the prevention of macrovascular disease^11^,^12^.

This contradictory evidence has led to the suggestion that elevated blood glucose does not cause CHD^13^, and if true, this would raise the possibility that the effects of T2D and FG seen in observational studies are largely due to confounding factors. The causality of the relationships between T2D, FG and CHD are of great importance to global public health, since the worldwide prevalence of T2D was 6.4% in 2010 (ref. 14) and CHD is the leading cause of death in the world^15^. Moreover, the total cost of T2D exceeded $174 billion in the US alone in 2007 (ref. 16).

Confounding may strongly influence observational studies, particularly when confounding factors are unknown or inadequately measured^17^. T2D and FG are closely associated with several of the mechanisms that lead to CHD independently of diabetes, such as increased body weight, LDL cholesterol, blood pressure^2^ and impaired endothelial function^18^. If these factors or unknown confounders influenced observational studies, then clinical practice guidelines recommending glucose-lowering therapy to prevent CHD and other macrovascular complications in diabetic patients should be reconsidered^11^,^19^. Unconfounded estimates of the relationship between T2D, related metabolic traits and CHD risk are therefore needed to better design and test interventions to reduce CHD risk in diabetic individuals.

Mendelian randomization (MR) is a study design in which genetic variants are employed as instrumental variables for estimating the unconfounded effect of an exposure (for example, T2D or FG) on a disease (for example, CHD)^20^. Although common genetic variants typically have only small effects on complex diseases, the combined use of multiple variants as instruments increases the statistical power to detect associations between exposure and outcome^21^,^22^,^23^. Because MR studies make use of the random assortment of alleles at meiosis, their estimates are much less vulnerable to confounding than observational epidemiologic studies. Furthermore, because allele assignment at meiosis precedes the onset of CHD, MR studies are not prone to reverse causation. Last, MR studies describe the effect of lifetime exposure to an allele, whereas RCTs assess the effect of an intervention, generally for less than a decade. For these reasons, when suitable genetic variants are available, MR studies can provide evidence in support of a causal association between exposure and outcome.

In the present study, we analyse summary-level genome-wide association study (GWAS) data from multiple genetic variants to obtain MR estimates of the effect of T2D and FG on CHD. We assess FG in non-diabetic subjects since FG in the non-diabetic range has previously been associated with CHD risk^2^, and since precise estimates of the effects of genetic variants on FG in non-diabetic subjects are available. We did not examine the effects of FG in diabetic subjects since drugs used to treat T2D influence FG. To obtain genetic variants that could serve as valid and independent instruments, we first search the largest available GWAS studies to date to identify single nucleotide polymorphisms (SNPs) significantly associated with T2D^24^,^25^,^26^ and FG^24^. We then test for and exclude from our analysis any of these candidate SNPs that either are in significant linkage disequilibrium with one another, or that are associated with known risk factors for CHD, including LDL cholesterol, triglycerides, systolic blood pressure, diastolic blood pressure and BMI. Using the remaining genetic variants as independent instruments, we apply statistical methods from meta-analysis to estimate the effect of T2D risk and FG levels on CHD risk. We then apply similar methodology to genetic variants associated with HbA1c. Finally, to examine the sources of heterogeneity in our estimate, we perform a subgroup analysis in which genetic variants are classified by their putative mechanism of action.

## Results

### T2D and CHD risk

**Figure 1:**
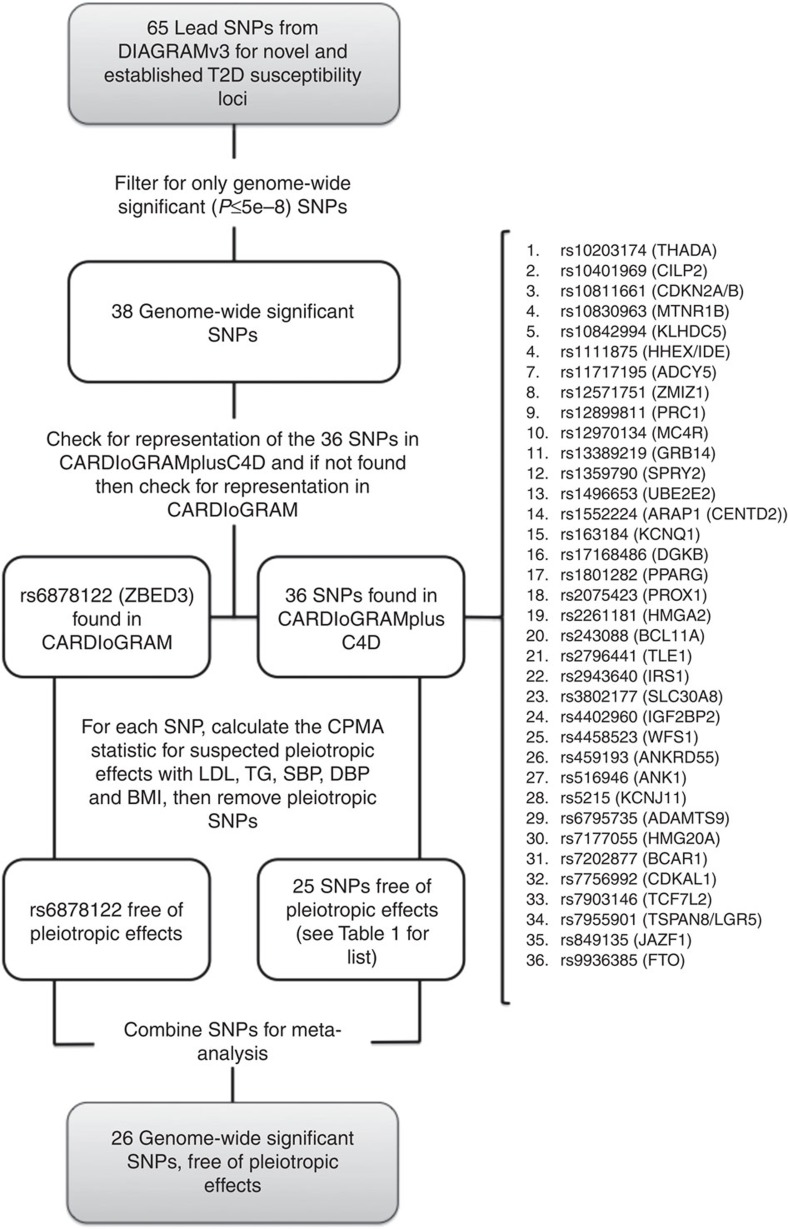
Selection and validation of T2D SNPs used as instruments in the Mendelian randomization analysis of the effect of T2D on CHD risk. .

To identify candidate instruments for assessing the effect of T2D on CHD risk, we found 38 genetic variants that had genome-wide significant (*P*<5 × 10^−8^ for the allelic effect of each SNP on T2D risk) associations with T2D in the largest GWA study to date (DIAGRAMv3, containing 34,840 cases and 114,981 controls)^25^, and assessed their effect on CHD risk in the largest GWA study of CHD to date (CARDIoGRAMplusC4D, containing 63,746 cases and 130,681 controls)^27^. If SNP results were absent from CARDIoGRAMplusC4D, their effects were drawn from CARDIoGRAM^28^, the second largest CHD GWAS to date. In all, 37 of 38 SNPs were assessed using either CARDIoGRAMplusC4D or CARDIoGRAM data. For 1 of the 38 variants (rs11651052 from the *HNF1B* [*TCF2*] locus), neither the index SNP nor any of its proxies (defined as variants with linkage disequilibrium *r*^2^>0.9 in HapMap CEU population) was found in either the CARDIoGRAMplusC4D or CARDIoGRAM dataset. This variant was thus excluded from further analyses. The set of 37 T2D risk-increasing variants for which both T2D and CHD data were available constituted the set of candidate variants, and SNPs in this set were further evaluated in our MR study (Table 1 and Fig. 1). We found through a random-effects meta-analysis of the 37 T2D risk-increasing candidate variants that the typical genome-wide significant T2D risk allele was associated with an odds ratio (OR) for risk of T2D of 1.11 (95% confidence interval (95% CI): 1.09–1.12) (Supplementary Table 1).

Since MR analysis requires that variants with known pleiotropy be excluded, we then tested the 37 candidate variants for association with known pleiotropic factors (including LDL-C, triglycerides systolic blood pressure, diastolic blood pressure and BMI) using cross-phenotype meta-analysis (CPMA), a statistical procedure for using summary-level GWAS data to identify pleiotropic associations among traits^29^ (Table 1 and Supplementary Table 2A). Of the 37 candidate variants, 11 were found to have potentially pleiotropic associations. Linkage equilibrium among the remaining 26 variants was confirmed using the HapMap dataset, accessed through the online tool SNAP^30^ (Fig. 1). The resulting set of 26 variants free of pleiotropic association with known risk factors and independent from one another by linkage constituted the MR-instrument set for T2D. Effect-size data for this set of non-pleiotropic variants provided the basis for our MR analysis.

We first carried out an analysis of instrumental-variables estimates derived from all 37 SNPs in the set of candidate variants, including the 11 SNPs with pleiotropic effects on serum lipid profiles, blood pressure and BM; this yielded an OR of 1.11 (95% CI: 1.05–1.16); *P*=1.7 × 10^−4^ for MR analyses; *I*^2^=63% (95% CI: 47–74%) (Table 2). Of the six SNPs in the set of candidate variants that were associated with the largest absolute effects on CHD risk, five were found to have pleiotropic associations with confounding risk factors. These pleiotropic SNPs included three with large positive effects (for example, *MC4R*, *IRS1* and *FTO*) and two with large negative effects (for example, *CILP2* and *ADAMTS9*) on CHD risk. Because of their pleiotropic associations, they were excluded from MR analysis (Table 1 and Supplementary Table 2A). As discussed below, the remaining SNP, near *BCAR1*, made a dominant contribution to the heterogeneity of the MR estimate assessed using the MR-instrument set.

**Figure 2:**
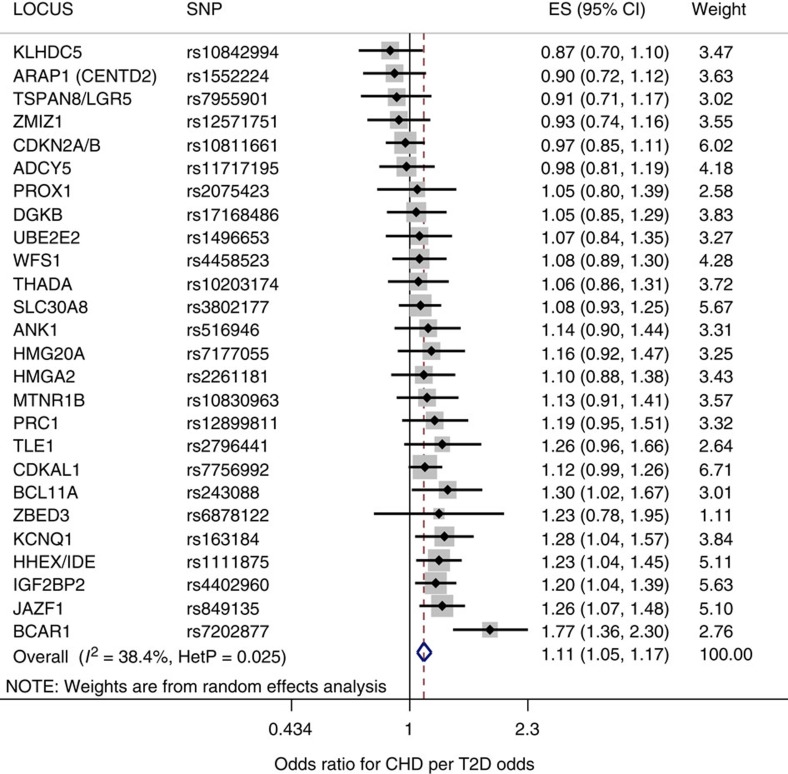
The Mendelian randomization estimate of the effect of T2D on CHD using a random-effects model. For each of the 26 non-pleiotropic SNPs (Table 1), the Forest plot shows the estimate of the effect of genetically increased T2D risk on CHD risk, as assessed for each SNP. Also shown for each SNP is the 95% confidence interval (black line segment) of the estimate and the inverse-variance weight (% proportional to the size of the grey square) in the random-effects meta-analysis.

To estimate the effect of genetically raised T2D risk on CHD risk, we used a random-effects model in which the 26 T2D risk-increasing variants of the MR-instrument set were treated as instrumental variables. (Table 1 and Methods section). A random-effects model derived from the MR-instrument set yielded a pooled MR estimate of the effect of T2D on CHD risk, and demonstrated that T2D increased the odds of CHD by 1.11 (95% CI: 1.05–1.17); *P*=8.8 × 10^−5^, *I*^2^=38 (95% CI: 1–62%) (Table 2, Fig. 2). Fixed-effects estimates for both the full set of candidate SNPs and for the set of MR instruments yielded results similar to those of the random-effects model (Supplementary Table 3).

To assess whether individual variants made large contributions to the heterogeneity of the MR estimate, we exhaustively computed and compared random-effects estimates of all subgroups of non-pleiotropic variants in the MR-instrument set, using a previously described combinatorial approach^31^. A single variant near *BCAR1* with a large effect on T2D risk dominated the heterogeneity in our MR estimate; removing this variant produced a risk estimate of markedly lower heterogeneity: (OR=1.09 (95% CI: 1.05–1.14); *P*=5.0 × 10^−5^ for MR analysis; *I*^2^=15% (95% CI: 0–48%)). Two other variants in the MR-instrument set (at the *KLDHC5* and *CDKN2A/B* loci) were found through exhaustive subgroup analysis to make important contributions to the heterogeneity in the MR estimate. Removing from the MR-instrument set the three variants associated with the *BCAR1, KLHDC5* and *CDKN2A/B* loci yielded a risk estimate with a heterogeneity estimate of zero. (OR=1.12 (95% CI: 1.07–1.16); *P*=2.7 × 10^−7^ for MR analysis; *I*^2^=0% (95% CI: 0–45%)).

### Biological subgroup analysis

**Figure 3:**
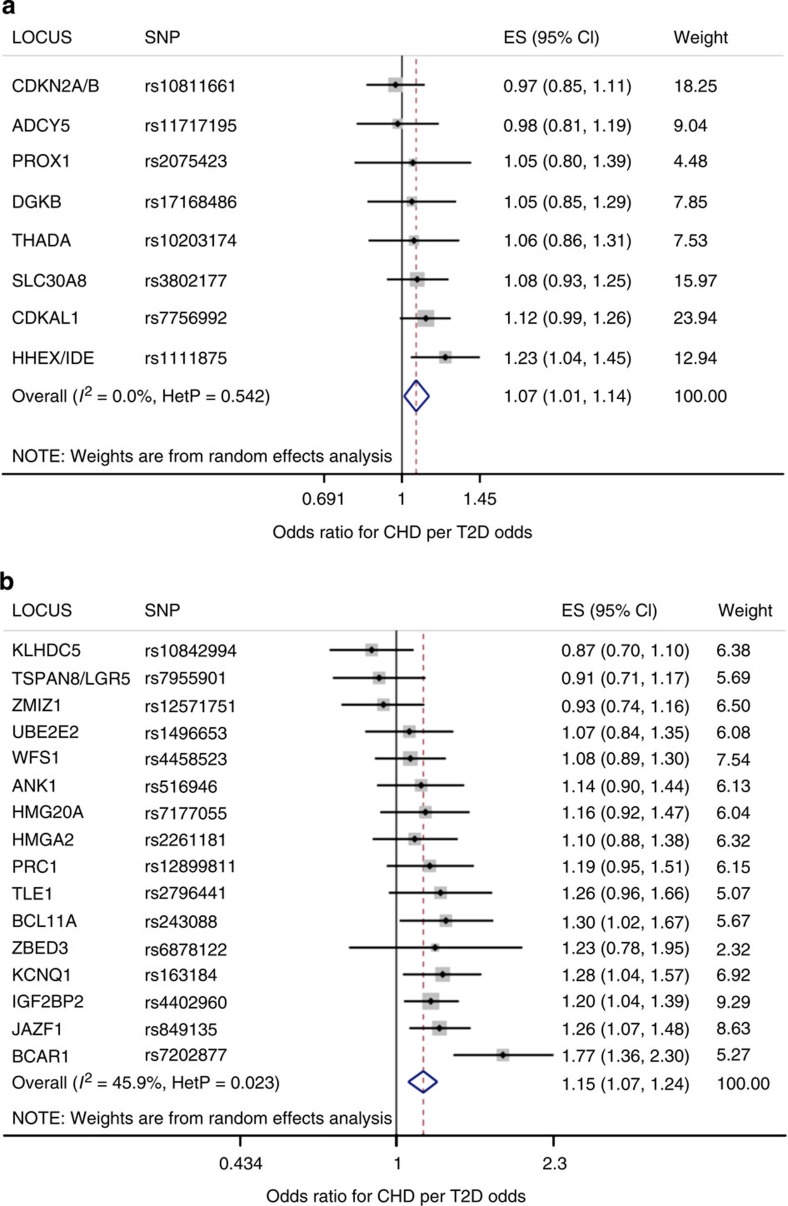
Mendelian randomization estimate of genetically increased T2D risk on CHD risk: subgroup analysis by physiologic cluster, computed using a random-effects model. Shown for each SNP is mean value (black sqaure), the 95% confidence interval (black line segment) of the estimate and the inverse-variance weight (% proportional to the size of the grey square) in the random-effects meta-analysis (blue diamond). Of five biologically distinct clusters of genetic variants, only two clusters contained enough significant, non-pleiotropic variants for further analysis: (**a**) the cluster of variants influencing beta-cell function; and (**b**) the cluster unclassified variants.

We next grouped T2D candidate variants based on their pathophysiologic mechanism, as proposed by a clustering analysis based on their genetic association with glycemic traits^32^. The candidate variants for which classification data were available and were placed in one of the five subgroups: (i) altered insulin sensitivity; (ii) reduced insulin secretion; (iii) defects in insulin processing; (iv) insulin secretion without a detectable changed in plasma glucose and (v) unclassified (Table 1). Importantly, all of the T2D variants associated with altered insulin sensitivity (*GCKR, IRS1, KLF14 and PPARG*) also influenced additional metabolic traits such as LDL cholesterol (Supplementary Table 4) and thus were not part of the MR-instrument set and were not included in the MR analysis. The pro-insulin cluster contained only one SNP, which was found to have pleiotropic effects; and the hyperglycemia cluster contains only two SNPs, one of which was found to have pleiotropic effects. After removing pleiotropic SNPs, there were only sufficient numbers of SNPs remaining to test the beta-cell subgroup and the unclassified subgroup. A random-effects analysis of the subgroup of the MR-instrument set associated with decreased beta-cell function (*N*=8) yielded an MR estimate of the effect of T2D on CHD risk of 1.07 (95% CI: 1.01–1.14); *P*=0.02 for MR analysis; *I*^2^=0% (95% CI: 0–68%)) and estimates based on T2D risk alleles with no clear effect on the above mechanisms (*N*=18) also conferred increased risk of CHD (OR=1.13 (1.04–1.23); *P*=3.4 × 10^−3^ for MR analysis; *I*^2^=60% (32–76%)) (Table 3 and Fig. 3).

### FG and CHD risk

**Figure 4:**
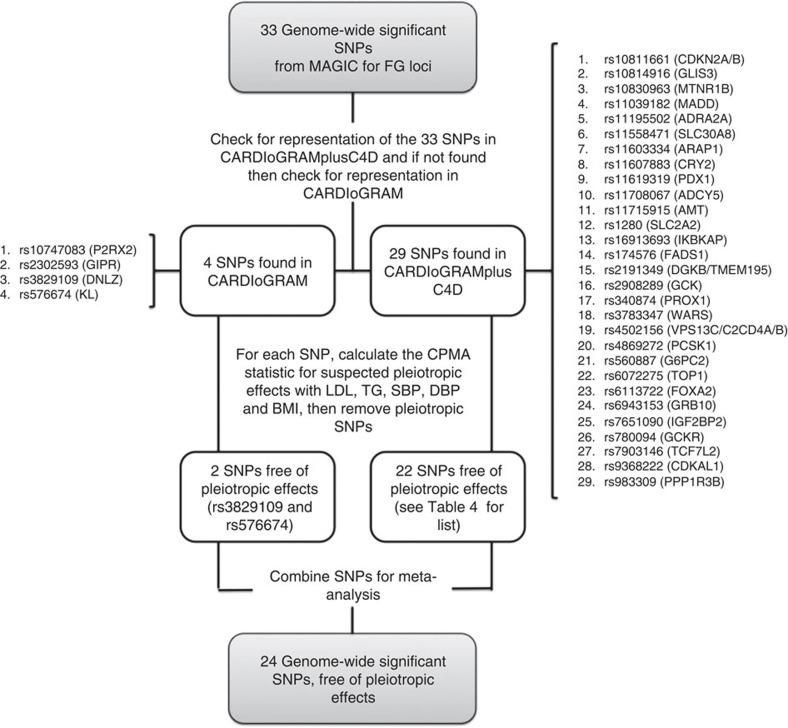
Selection and validation of fasting glucose SNPs used as instruments in the Mendelian randomization analysis of the effect of fasting glucose on CHD risk.

We identified 33 variants that showed genome-wide significant (*P*<5 × 10^−8^) associations with FG levels (Table 4 and Fig. 4) using data from the MAGIC consortium's most recent GWAS for FG, which included 133,010 non-diabetic individuals^24^. Just as for the analysis of T2D candidate variants described above, the effects of the FG variants on CHD risk were ascertained in the largest GWAS to date for CHD^27^, and if unavailable in the second largest GWAS to date for CHD^28^ (Fig. 4). Data on CHD for all 33 SNPs were available, and thus all 33 SNPs were included in the set of candidate variants for FG. We found through a random-effects meta-analysis of this set of FG-increasing candidate variants that a typical genome-wide significant FG risk allele was associated with a 0.028 mmol l^−1^ increase in FG (95% CI: 0.021–0.035 mmol l^−1^) (Supplementary Note 1 and Supplementary Table 1).

Just as for T2D risk alleles, we used the CPMA statistic to test the FG candidate variants for pleiotropic associations with LDL-C, triglycerides, systolic blood pressure, diastolic blood pressure and BMI (Table 4 and Supplementary Table 2B). Of these 33 candidate SNPs, 9 were found to have pleiotropic effects. Just as for the T2D analysis described above, all FG candidate variants were confirmed to be in pairwise linkage equilibrium. The remaining 24 non-pleiotropic, independent variants constituted the MR-instrument set for FG, and were used to compute the MR estimate for the effect of FG on CHD.

**Figure 5:**
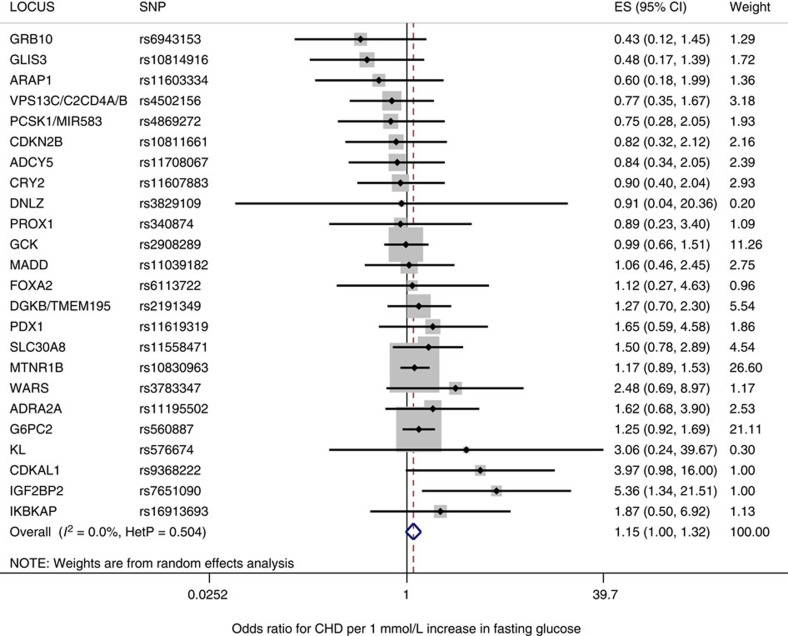
The Mendelian randomization estimate of the effect of fasting glucose on CHD using a random-effects model. For each of the 24 non-pleiotropic SNPs (Table 4), the Forest plot shows the estimate of the effect of the Fasting Glucose risk allele upon CHD risk, as assessed for each SNP, the 95% confidence interval (black line segment) of the estimate and the inverse-variance weight (proportional to the size of the grey square) in the random-effects meta-analysis (blue diamond).

A random-effects analysis using the full set of 33 candidate variants yielded an effect-size estimate of 1.27 CHD odds per 1 mmol l^−1^ increase in FG (95% CI: 1.04–1.54 CHD odds per 1 mmol l^−1^ increase in FG); *P*=0.02 for MR analysis; *I*^2^=39% (7–60%) (Table 2). The effect of FG on CHD, as measured using the 24 FG risk alleles of the MR-instrument set for FG, was 1.15 CHD odds per 1 mmol l^−1^ FG (95% CI: 1.00–1.32 CHD odds per 1 mmol l^−1^ increase in FG); *P*=0.05 for MR analysis, *I*^2^=0% (0–44.6%) (Table 2 and Fig. 5).

To assess whether related glycemic traits in non-diabetics also were causally associated with increased risk of CHD, we undertook a similar analysis of HbA1c levels on CHD risk, which did not yield statistically significant results (Supplementary Note 2).

## Discussion

Using summary-level data for T2D and FG risk alleles, our MR study supports observational evidence, suggesting that T2D and FG lead to CHD. These findings have important implications both for the care of T2D patients, and for the design of clinical trials to assess the effect of T2D treatments on cardiovascular risk; they support the hypothesis that lowering T2D risk and glucose levels can help prevent CHD. While these findings contrast with recent short-term RCTs investigating the effect of glucose lowering in T2D^5^,^6^,^7^,^8^, an important difference between MR studies and RCTs is that MR studies describe the effect of a lifetime of exposure to glucose lowering alleles in the general population, whereas RCTs measure the short-term effects (that is, <7 years) of intensive glucose-lowering therapy on CHD risk in patients with diabetes (and therefore with established hyperglycemia)^5^,^6^,^7^,^8^. While it is possible that RCTs designed to test glucose lowering may need substantially longer follow-up times to fully estimate the effect of these interventions on CHD, our data does not permit direct insights into this hypothesis, and other mechanisms could be responsible for the lack of clear effect demonstrated in RCTs to date (such as the mechanisms by which glucose is lowered, or potential adverse cardiovascular effects of some of the T2D treatments used in clinical trials).

Importantly, almost all current treatments for T2D focus on lowering glucose levels. Our study considered the effects of FG on CHD for individuals without T2D. However, since the effect of FG on CHD risk is considerably higher among diabetic individuals^2^, the observed effect for FG in this study may underestimate the effect of genetically elevated glucose levels in individuals with T2D. To our knowledge, this hypothesis is not testable at present, since there are currently an insufficient number of genetic variants robustly associated with FG among diabetic individuals. Nonetheless, we find that even a small increase in genetically elevated FG levels in non-diabetic individuals is associated with a trend towards increased CHD risk. This result suggests that glucose is an important mediator linking T2D and CHD pathogenesis.

A relatively small proportion (4.8%) of the variance in FG levels in the non-diabetic population is explained by the common genetic variants used in this study^24^. Our use of multiple variants in the MR analysis increases the statistical power to detect causal associations, although at the expense of increased finite sample bias^21^. The variance explained by genetic factors will likely increase as the number of individuals participating in large-scale GWAS increases and as results from large-scale whole-genome and whole-exome sequencing studies identify additional genetic variants associated with elevated blood glucose levels. Furthermore, as genetic factors explain a greater proportion of the variance in FG, the error in the associated MR estimates of the effect of FG on CHD would be expected to decrease. This is because MR estimates which use instrumental variables explaining little variance in a trait tend to be biased towards the null^17^. Such bias is unlikely to have influenced either the direction or significance of the results of this study since our MR analysis shows a positive relationship between FG and CHD. We note that FG may have a non-linear relationship with CHD, but only for individuals with low glucose levels, which represents a minority of the general population^2^. Last, results from our MR of HbA1c levels may have been biased towards the null since many HbA1c variants are clearly non-glycemic^24^.

A strength of our study is that data on associations between exposure, outcome and confounder traits were typically gathered in different population samples; this approach reduces the possibility of over-fitting effect-size estimates. Moreover, the fact that we draw effect-size data from separate large-scale GWA studies for exposure and outcome traits means that effect sizes are more precisely assessed than would be possible by the analysis of individual-level data from a smaller study.

Although this has the potential to introduce error because variation in risk exposure between study populations can distort the estimated effect, this error is expected to be small since the various studies that were part of the DIAGRAMv3, CARDIoGRAM and MAGIC meta-analyses often drew subjects from the same overall population samples. Several factors can lead to bias in our estimate of the effect of genetically elevated risk of T2D and FG^22^,^23^. Although we checked for pleiotropic associations with major known confounders, there may be associations with unknown confounders leading to bias. A further complication arises from feedback interactions (such as canalization) and other non-linear interactions between the exposure and its confounders. Canalization, the process by which compensatory feedback mechanisms reduce the phenotypic consequences of genetic variation, has been extensively studied in the context of MR (reviewed elsewhere)^33^,^34^,^35^. However, since canalization tends to bias results towards the null, the presence of canalization would not alter the statistical significance or direction of the effects we detect through MR.

MR has previously been used to show that non-FG in non-diabetic individuals is causally related to ischaemic heart disease^36^. This previous study used individual-level data from a large group of Danish subjects, and its findings on the effect of non-FG levels are consistent with our findings on FG levels. An additional study showed that variants that predict CHD risk were significantly associated with related metabolic phenotypes in a population of 1,208 diabetic subjects, but did not directly assess whether T2D risk variants were associated with risk of CHD^37^.

In addition to non-fasting blood glucose, numerous other traits have been assessed through MR studies for their impact on cardiovascular risk^38^, including traits related to glucose metabolism^36^,^38^. Some appear to be biomarkers without substantial causal influence: HDL^39^, CRP^40^, homocysteine^41^, bilirubin^42^ and uric acid^43^. Other traits appear to be causally related, including hypertension^44^, lipid metabolism such as LDL^45^, Lp(a)^46^, triglycerides^47^,^48^ and adiposity^49^. While adiponectin shares an allelic architecture with CHD^50^, this is likely due to pleiotropic effects^51^. Our study provides evidence that T2D, like LDL and obesity, has a causal effect on CHD; and that this effect is discernible even after correcting for pleiotropic associations with known confounding factors.

In this study, we used MR analysis of summary-level GWAS data to provide evidence that genetically increased risk of T2D leads to increased CHD risk. We also provide evidence through MR analysis for a trend, indicating that increases in FG in non-diabetics leads to an increase in CHD risk. Our results support the hypothesis that the relationship between T2D and CHD is indeed causal, and that associated metabolic traits in non-diabetics may also have a causal influence on CHD risk. These findings suggest that long-term efforts to prevent T2D and lower glucose levels can decrease CHD risk.

## Methods

### Candidate instrument selection

We gathered data from large meta-analyses of GWA studies examining the exposure (T2D and FG), outcome (CHD) and confounder traits (specifically: LDL cholesterol, triglycerides, systolic blood pressure, diastolic blood pressure and BMI) from the largest GWAS studies to date^21^,^22^,^23^. We used as our initial set of instrumental variables the 38 genome-wide significant (*P* value≤5 × 10^−8^) SNPs associated with increased T2D risk identified in the DIAGRAM consortium, the largest meta-analysis to date of the T2D GWAS studies^25^. The DIAGRAM meta-analysis includes data from 34,840 T2D cases and 114,981 controls of predominantly European descent^25^. Allele frequencies for this meta-analysis were drawn from the 1000 Genomes dataset, and linkage disequilibrium was calculated using CEU linkage data.

We gathered GWAS data on FG from the largest meta-analysis to date (carried out by the MAGIC consortium)^24^ of GWA studies examining the genetic architecture of glycemic traits in non-diabetic individuals. For FG, 33 independent genome-wide significant SNPs were selected.

For each of the susceptibility variants for T2D and FG, we sought summary-level data for CHD from the CARDIoGRAMplusC4D Metabochip study, since this is the largest GWAS meta-analysis for CHD to date^27^. This study profiled ∼200,000 SNPs contained in loci previously associated with cardiometabolic trait expression or disease risk in 63,746 cases and 130,681 controls of predominantly European descent. Since the Metabochip has limited SNP content, summary-level data for SNPs in the T2D or FG reference sets that were not genotyped in the CARDIoGRAMplusC4D Metabochip study were obtained from the CARDIoGRAM GWAS, which was the next largest study and comprised 22,233 cases and 64,762 controls^28^ (Fig. 1). We verified through an analysis of the demographic data available for the CARDIoGRAMplusC4D study that the exposure to T2D for individuals enroled in this study was of sufficient duration for an MR analysis to provide a reliable estimate of the effect of T2D on CHD (Supplementary Note 3).

In all, 37 of 38 significant T2D variants were represented either in the CARDIoGRAMplusC4D or CARDIoGRAM datasets. One of the 38 T2D variants, namely rs11651052, was absent from both the CARDIoGRAMplusC4D and the CARDIoGRAM datasets. Moreover, no variant in close linkage disequilibrium with rs11651052 could serve as a proxy for it in our analysis. For this reason, we excluded it from further analysis. The remaining set of 37 candidate variants provided the basis for our analysis of the effect of T2D on CHD, and contains most of the lead variants used previously in genetic risk scores for predicting the risk of T2D (refs 52, 53). All 33 significant FG variants were represented in the outcome datasets, and thus all were further evaluated for inclusion in our MR analysis.

For pleiotropic traits, summary-level results were similarly sourced from the largest GWAS conducted to date for each trait: (i) LDL-C^54^, (ii) triglycerides^54^, (iii) systolic blood pressure, (iv) diastolic blood pressure^55^ and (v) BMI^56^. Cohorts contributing to these pleiotropic traits were largely population based^54^,^55^,^56^. Linkage equilibrium of all variants was assessed using SNAP^30^ applied to the HapMap European samples.

### Candidate instrument validation

We assessed each SNP for evidence of pleiotropic associations using an omnibus test on *P* values called CPMA^29^. The CPMA test compares the observed distribution of *P* values across phenotypes to that predicted by the null hypothesis of no pleiotropic association, under which *P* values are uniformly distributed. Variants with any detectable association with pleiotropic traits were removed from the analysis, and the remaining non-pleiotropic variants were taken as instruments for the MR analysis (Supplementary Table 2A,B).

The CPMA approach to screening for pleiotropic associations has several limitations. First, it is possible that variants have pleiotropic effects that are not detected by the CPMA test. Further data than is currently available from publicly accessible GWAS datasets may be necessary to detect some associations. Second, the CPMA method treats every statistically significant association with a potentially pleiotropic pathway as a true instance of pleiotropy. This approach is conservative in that it excludes any variant for which there is statistical evidence of potentially pleiotropic effects, independent of the strength or the direction of such effects. Although it is possible that some non-pleiotropic variants may be excluded by this method, removing such variants from the MR analysis will favour the null hypothesis of no association between exposure and outcome. Consequently, an MR analysis that uses this procedure and yields statistically significant results is likely to reflect a true causal association between exposure and outcome.

### Statistical analysis of instrumental-variable estimates

For each instrument, we obtained an estimate of the effect of the exposure on the outcome using summary-level data. Let *x* and *y* denote the centred and scaled exposure and outcome traits, respectively, and these are related by the linear structural equation: *y=αx+η*. Here *η* is a stochastic error term, and in general *x* and *η* are correlated because of confounding. The parameter *α* quantifies the causal effect of *x* on *y*, and is thus the parameter we seek to estimate. Let *u*_*i*_ denote the allele dosage variable of the *i*th genetic variant. Let *γ*_*i*_ and *β*_*i*_ denote effect-size estimates (derived from GWAS data) of *u*_*i*_ on the exposure *x* and outcome *y,* respectively, and let *s(β*_*i*_) denote the s.e. of *β*_*i*_. Then the MR estimate associated with the *i*th genetic variant is:


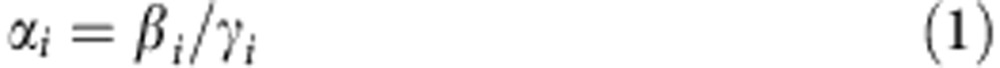


and the variance of this estimate is:


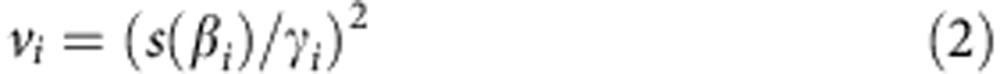


Define the precision of the *i*th MR estimate of *α* by *w*_*i*_*=1/v*_*i*_. The inverse-variance-weighted fixed-effects estimate is then:


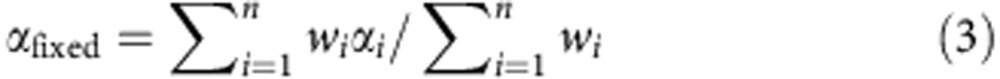


and the s.e. *s(α*_fixed_) of this estimate is given by


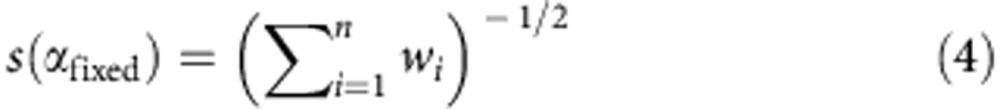


We observe that *α*_fixed_ may also be interpreted as the regression coefficient resulting from the generalized linear regression of the outcome effect size *β*_*i*_ on the exposure effect size *γ*_*i*_ assuming heteroskedastic errors; in this regression, the *i*th error term has a variance equal to *s(β*_*i*_)^*2*^, and the offset coefficient in the regression is zero.

The random-effects estimate *α*_random_ estimate and its s.e. *s(α*_random_) are constructed from the individual estimates using standard methods^57^, in which the weights are adjusted to account for the intrinsic variability (or heterogeneity) in the effect size. Heterogeneity may be quantified in the random-effects model with the parameter *I*^2^, which reports the fraction of the total variance in the meta-analytic estimate that is due to intrinsic variability in the effect size, as distinct from the variability arising due to measurement error^58^. The random-effects estimate *α*_random_ and its s.e. *s(α*_random_) are given by equations analogous to those for *α*_fixed_ and *s(α*_fixed_), in which the weights assigned to individual estimates are adjusted to take into account heterogeneity in the effect size.

For each of the exposure traits (T2D and FG), we carried out a meta-analysis of estimates obtained from individual susceptibility variants using both fixed-effects and random-effects models to obtain pooled estimates of the effect of exposure on outcome. Such meta-analytic methods are commonly used to summarize information from independent studies for the effect of an intervention on a health outcome. In this study design, we use independent genetic variants as instruments to assess the effect of exposure (T2D or FG) on outcome (CHD), and then pool these individual estimates using statistically efficient estimators formally analogous to those of inverse-variance-weighted meta-analysis^31^.

For each of the exposure traits, we also carry out a random-effects meta-analysis. Because the heterogeneity in the effect sizes for individual SNPs is large for each of the exposures, our random-effects estimates are close to an unweighted average of the effect sizes.

For all MR meta-analyses, we report estimates from the random-effects models (in the main text and in Table 2) and fixed-effects models (Supplementary Table 3). The effect-sizes for each meta-analysis is reported as the OR describing the effect of the exposure on the outcome, given by exp*(α*_fixed_) for the fixed-effects model and by exp*(α*_random_) for the random-effects model.

### Subgroup analyses

*Combinatorial analysis of heterogeneity*. Heterogeneity of effects on risk of CHD may be observed and may point to pathways that have disparate effects on risk of T2D and CHD. To determine which SNPs are responsible for heterogeneity measured by *I*^2^, we undertook a sensitivity analysis using a combinatorial approach^31^, in which the heterogeneity indicators were computed for risk scores in which we exhaustively computed effect size and heterogeneity estimates for groups of variants in which combinations of SNPs were excluded^31^.

We carried out a heterogeneity analysis both of the pooled effect-size estimates derived from the full set of variants and of set of variants used for MR analysis, from which pleiotropic SNPs were excluded. The purpose of this kind of analysis is to determine whether one or a small number of variants contribute heavily to the effect-size heterogeneity estimates^31^.

*Biological subgroups of T2D variants*: To assess the sources of heterogeneity in the pooled estimates, we carried out a subgroup analysis using an approach that clusters instruments according to the mechanisms through which they act on risk of T2D. We used a classification of T2D SNPs proposed by Dimas *et al*.^32^, based on a cluster analysis of their associations with related metabolic traits. Under this clustering, T2D risk alleles were assigned to one of the five categories according to the association between their corresponding loci and (i) altered insulin sensitivity; (ii) reduced insulin secretion; (iii) defects in insulin processing; (iv) insulin secretion without a detectable changed in plasma glucose; (v) no clear association with glycemic traits (Supplementary Table 4). This analysis permits an assessment of the mechanistic pathways through which T2D genetic variants may impact risk of CHD. As expected all four SNPs in the insulin resistance subgroup delineated by Dimas *et al*.^32^, at loci (*GCKR, IRS1, KLF14* and *PPARG*) had pleiotropic effects (Table 1, Supplementary Table 4) and were thus excluded from our main analysis. A large proportion of the previously classified SNPs had *P* values below the genome-wide significant threshold (Supplementary Table 4), and consequently were also excluded from further analysis. After excluding non-significant and pleiotropic SNPs only two subgroups remained: beta-cell and unclassified variants.

## Additional information

**How to cite this article:** Ahmad, O. S. *et al*. A Mendelian randomization study of the effect of type-2 diabetes on coronary heart disease. *Nat. Commun*. 6:7060 doi: 10.1038/ncomms8060 (2015).

## Supplementary Material

Supplementary Information Supplementary Figures 1-5, Supplementary Tables 1-7, Supplementary Notes 1-3 and Supplementary References

## Figures and Tables

**Table 1:** Characteristics of SNPs considered for use in Mendelian randomization analysis of the effect of T2D on CHD risk.

Locus	SNP	EA	NEA	OR T2D	*P* value T2D	OR CHD	*P* value CHD	Pleiotropic effect	Physiologic cluster
ADCY5	rs11717195	T	C	1.11	6.5E−14	1.00	8.3E−01	No	BC
CDKAL1	rs7756992	G	A	1.17	7.0E−35	1.02	7.2E−02	No	BC
CDKN2A/B	rs10811661	T	C	1.18	3.7E−27	1.00	6.8E−01	No	BC
DGKB	rs17168486	T	C	1.11	5.9E−11	1.00	6.6E−01	No	BC
HHEX/IDE	rs1111875	C	T	1.11	2.0E−19	1.02	1.3E−02	No	BC
PROX1	rs2075423	G	T	1.07	8.1E−09	1.00	7.1E−01	No	BC
SLC30A8	rs3802177	G	A	1.14	1.3E−21	1.01	3.3E−01	No	BC
THADA	rs10203174	C	T	1.14	9.5E−12	1.01	5.9E−01	No	BC
MTNR1B	rs10830963	G	C	1.10	5.3E−13	1.01	2.6E−01	No	HG
ANK1	rs516946	C	T	1.09	2.5E−10	1.01	2.8E−01	No	NA
BCAR1	rs7202877	T	G	1.12	3.5E−08	1.07	2.7E−05	No	NA
HMG20A	rs7177055	A	G	1.08	4.6E−09	1.01	2.1E−01	No	NA
KLHDC5	rs10842994	C	T	1.10	6.1E−10	0.99	2.4E−01	No	NA
TLE1	rs2796441	G	A	1.07	5.4E−09	1.02	9.5E−02	No	NA
UBE2E2	rs1496653	A	G	1.09	3.6E−09	1.01	5.8E−01	No	NA
ZMIZ1	rs12571751	A	G	1.08	1.0E−10	0.99	5.2E−01	No	NA
ARAP1 (CENTD2)	rs1552224	A	C	1.11	1.8E−10	0.99	3.4E−01	No	PI
BCL11A	rs243088	T	A	1.07	1.8E−08	1.02	3.8E−02	No	UC
HMGA2	rs2261181	T	C	1.13	1.2E−09	1.01	4.0E−01	No	UC
IGF2BP2	rs4402960	T	G	1.13	2.4E−23	1.02	1.5E−02	No	UC
JAZF1	rs849135	G	A	1.11	3.1E−17	1.02	5.8E−03	No	UC
KCNQ1	rs163184	G	T	1.09	1.2E−11	1.02	2.2E−02	No	UC
PRC1	rs12899811	G	A	1.08	6.3E−09	1.01	1.4E−01	No	UC
TSPAN8/LGR5	rs7955901	C	T	1.07	6.5E−09	0.99	4.6E−01	No	UC
WFS1	rs4458523	G	T	1.10	2.0E−15	1.01	4.6E−01	No	UC
ZBED3	rs6878122	G	A	1.10	5.0E−11	1.02	3.8E−01	No	UC
TCF7L2	rs7903146	T	C	1.39	1.2E−139	1.03	5.7E−03	Yes	BC
IRS1	rs2943640	C	A	1.10	2.7E−14	1.03	5.2E−04	Yes	IR
PPARG	rs1801282	C	G	1.13	1.1E−12	1.00	7.8E−01	Yes	IR
ANKRD55	rs459193	G	A	1.08	6.0E−09	1.02	2.5E−02	Yes	NA
CILP2	rs10401969	C	T	1.13	7.0E−09	0.93	7.3E−05	Yes	NA
FTO	rs9936385	C	T	1.13	2.6E−23	1.03	4.2E−03	Yes	NA
GRB14	rs13389219	C	T	1.07	1.0E−08	1.02	7.2E−02	Yes	NA
MC4R	rs12970134	A	G	1.08	1.2E−08	1.03	1.8E−03	Yes	NA
SPRY2	rs1359790	G	A	1.08	1.4E−08	0.99	4.7E−01	Yes	NA
ADAMTS9	rs6795735	C	T	1.08	7.4E−11	0.98	1.8E−02	Yes	UC
KCNJ11	rs5215	C	T	1.07	8.5E−10	1.02	1.8E−02	Yes	UC

BC, beta-cell dysfunction; CHD, coronary heart disease; CPMA, cross-phenotype meta-analysis; EA, effect allele; HG, hyperglycemic; IR, insulin resistance; NA, not available; NEA, non-effect allele; OR, odds ratio; PI, pro-insulin; SNP, single nucleotide polymorphism; T2D, type-2 diabetes; UC, unclassified.

Pleiotropic effect: ‘Yes' indicates that the SNP was associated with at least one confounding trait in the CPMA analysis. See Supplementary Table 2 for a full description of these pleiotropic associations. Physiologic Clusters: UC, IR, BC, PI, HG and NA. Note that the OR for CHD is not weighted for the effect of each SNP on T2D or fasting glucose. Figures report the OR for CHD weighted by their effect on T2D.

**Table 2:** Random-effects model estimates for the effect of T2D and fasting glucose on CHD risk.

Instrument set	Effect size (95% CI)	*P* value	*I*^2^ (95% CI)
T2D (all significant SNPs, *n*=37)	1.11 (1.05–1.16)	1.7E−04	62.7 (46.8–73.8)
T2D (excluding pleiotropic SNPs, *n*=26)	1.11 (1.05–1.17)	8.8E−05	38.4 (1.2–61.6)
Fasting glucose (all significant SNPs, *n*=33)	1.27 (1.04–1.54)	2.0E−02	39.1 (7.3–60.0)
Fasting glucose excluding pleiotropic SNPs, *n*=24)	1.15 (1.00–1.32)	5.3E−02	0 (0–44.6)

CHD, coronary heart disease; CI, confidence interval; MR, Mendelian randomization; SNP, single nucleotide polymorphism; T2D, type-2 diabetes.

Effect size for T2D analysis is the effect on odds of CHD per odds increase in risk of T2D. Effect size for fasting glucose analysis is the effect on odds of CHD per 1 mmol l^−1^ increase in fasting glucose in non-diabetics. Each *P* value is for the MR analyses.

**Table 3:** Mendelian randomization estimate of effect of T2D on CHD risk for subgroup analyses of SNP physiologic clusters.

Cluster name	Effect size (95% CI)	*P* value	*I*^2^ (95% CI)
No cluster	1.13 (1.04–1.23)	3.4E−03	59.5 (32.0–75.9)
Beta-cell cluster	1.07 (1.01–1.14)	2.3E−02	0 (0–67.6)

CHD, coronary heart disease; CI, confidence interval; MR, Mendelian randomization; SNP, single nucleotide polymorphism; T2D, type-2 diabetes.

Each *P* value is for the MR analyses.

**Table 4:** Characteristics of SNPs considered for use in Mendelian randomization analysis of the effect of fasting glucose on CHD risk.

Locus	SNP	EA	NEA	Effect on FG (mmol l^−1^)	OR FG	*P* value FG	OR CHD	*P* value CHD	Pleiotropic effect
ADCY5	rs11708067	A	G	0.02	1.02	1.3E−18	1.00	7.0E−01	No
ADRA2A	rs11195502	C	T	0.03	1.03	2.0E−18	1.02	2.8E−E−01	No
ARAP1	rs11603334	G	A	0.02	1.02	1.1E−11	0.99	4.0E−01	No
CDKAL1	rs9368222	A	C	0.01	1.01	1.0E−09	1.02	5.3E−02	No
CDKN2B	rs10811661	T	C	0.02	1.02	5.7E−18	1.00	6.8E−01	No
CRY2	rs11607883	G	A	0.02	1.02	6.3E−24	1.00	8.1E−01	No
DGKB/TMEM195	rs2191349	T	G	0.03	1.03	1.3E−42	1.01	4.3E−01	No
DNLZ	rs3829109	G	A	0.02	1.02	1.1E−10	1.00	9.5E−01	No
FOXA2	rs6113722	G	A	0.04	1.04	2.5E−11	1.00	8.8E−01	No
G6PC2	rs560887	C	T	0.07	1.07	1.4E−178	1.02	1.5E−01	No
GCK	rs2908289	A	G	0.06	1.06	3.3E−88	1.00	9.8E−01	No
GLIS3	rs10814916	C	A	0.02	1.02	2.3E−13	0.99	1.7E−01	No
GRB10	rs6943153	T	C	0.02	1.02	1.6E−12	0.99	1.7E−01	No
IGF2BP2	rs7651090	G	A	0.01	1.01	1.8E−08	1.02	1.8E−02	No
IKBKAP	rs16913693	T	G	0.04	1.04	3.5E−11	1.03	3.5E−01	No
KL	rs576674	G	A	0.02	1.02	2.3E−8	1.02	3.9E−01	No
MADD	rs11039182	T	C	0.02	1.02	4.8E−22	1.00	8.9E−01	No
MTNR1B	rs10830963	G	C	0.08	1.08	1.1E−215	1.01	2.6E−01	No
PCSK1/MIR583	rs4869272	T	C	0.02	1.02	1.0E−15	0.99	5.8E−01	No
PDX1	rs11619319	G	A	0.02	1.02	1.3E−15	1.01	3.4E−01	No
PROX1	rs340874	C	T	0.01	1.01	4.1E−10	1.00	8.7E−01	No
SLC30A8	rs11558471	A	G	0.03	1.03	7.8E−37	1.01	2.2E−01	No
VPS13C/C2CD4A/B	rs4502156	T	C	0.02	1.02	1.4E−25	0.99	5.0E−01	No
WARS	rs3783347	G	T	0.02	1.02	1.3E−10	1.02	1.7E−01	No
AMT	rs11715915	C	T	0.01	1.01	4.9E−08	1.05	6.3E−06	Yes
FADS1	rs174576	C	A	0.02	1.02	1.2E−18	1.02	8.9E−02	Yes
GCKR	rs780094	C	T	0.03	1.03	2.6E−37	1.01	5.4E−01	Yes
GIPR	rs2302593	C	G	0.01	1.01	9.3E−10	1.03	1.2E−01	Yes
P2RX2	rs10747083	A	G	0.01	1.01	7.6E−09	1.02	4.2E−01	Yes
PPP1R3B/LOC157273	rs983309	T	G	0.03	1.03	6.3E−15	1.00	7.9E−01	Yes
SLC2A2	rs1280	T	C	0.03	1.03	8.6E−18	1.01	6.4E−01	Yes
TCF7L2	rs7903146	T	C	0.02	1.02	2.7E−20	1.03	5.7E−03	Yes
TOP1	rs6072275	A	G	0.02	1.02	1.7E−08	0.99	3.4E−01	Yes

CHD, coronary heart disease; CPMA, cross-phenotype meta-analysis; EA, effect allele; FG, fasting glucose; NEA, non-effect allele; OR, odds ratio; SNP, single nucleotide polymorphism; T2D, type-2 diabetes.

Pleiotropic Effect: ‘Yes' indicates that the SNP was associated with at least one confounding trait in the CPMA analysis. See Supplementary Table 2 for a full description of these pleiotropic associations. Note that the OR for CHD is not weighted for the effect of each SNP on fasting glucose. Figures report the OR for CHD weighted by their effect on fasting glucose.
