# Supplementary material for: A Mendelian randomization study of the effect of type-2 diabetes on coronary heart disease
Source: Nat Commun. 2015 May 28;6:7060. doi: 10.1038/ncomms8060 (PMC4458864; doi:10.1038/ncomms8060)
Supplement: Supplementary Information — Supplementary Figures 1-5, Supplementary Tables 1-7, Supplementary Notes 1-3 and Supplementary References [file ncomms8060-s1.pdf]

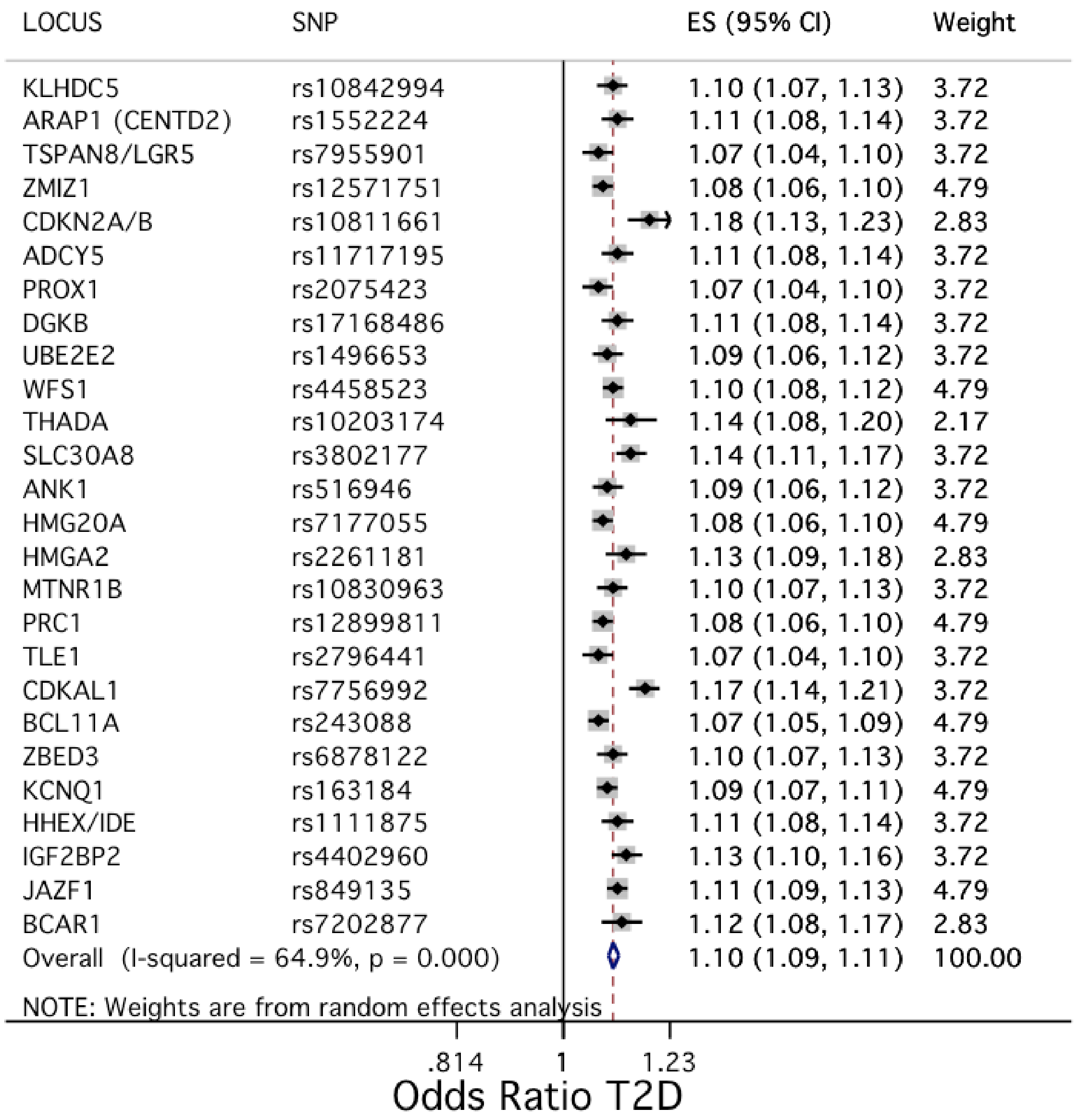

Supplementary Figure 1. Random-effects meta-analysis of T2D risk alleles upon risk of T2D. Pleiotropic SNPs (Table 1) have been removed. Shown for each SNP is the 95% confidence interval (black line segment) of the estimate and the inverse-variance weight (% proportional to the size of the grey square) in the random-effects meta-analysis.

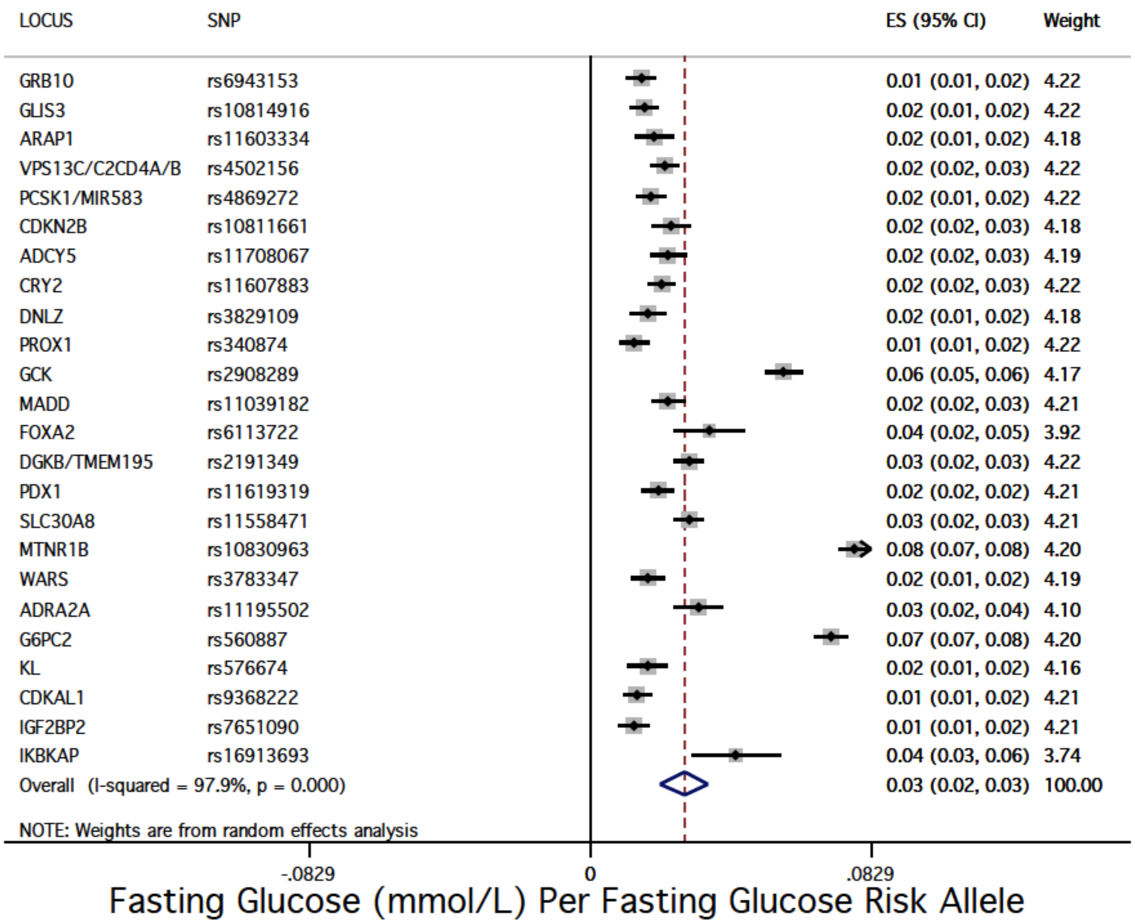

Supplementary Figure 2. Random-effects meta-analysis of fasting glucose risk alleles for their effect on fasting glucose. Pleiotropic SNPs (Table 2) have been removed. Also shown for each SNP is the 95% confidence interval (black line segment) of the estimate and the inverse-variance weight (% proportional to the size of the grey square) in the random-effects meta-analysis.

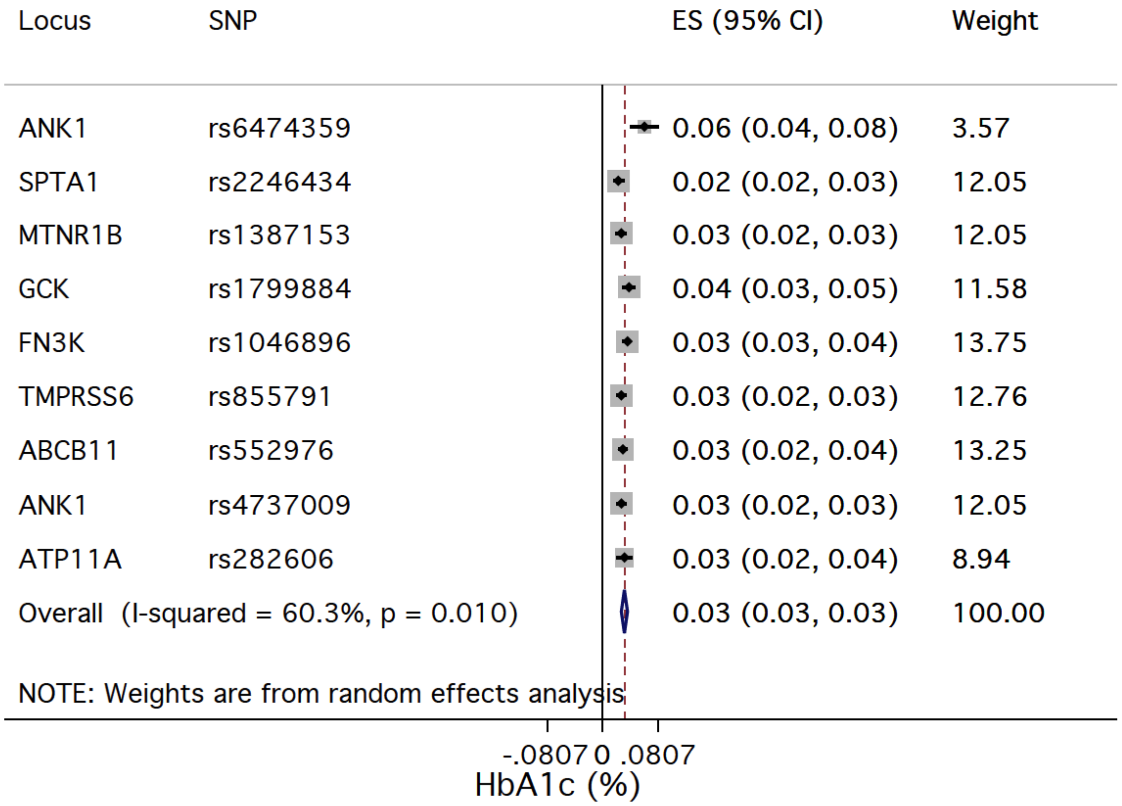

Supplementary Figure 3. Random-effects meta-analysis of hemoglobin A1c (HbA1c) risk alleles for their effect on HbA1c. Pleiotropic SNPs (Supplementary Table 5) have been removed. Also shown for each SNP is the 95% confidence interval (black line segment) of the estimate and the inverse-variance weight (% proportional to the size of the grey square) in the random-effects meta-analysis.

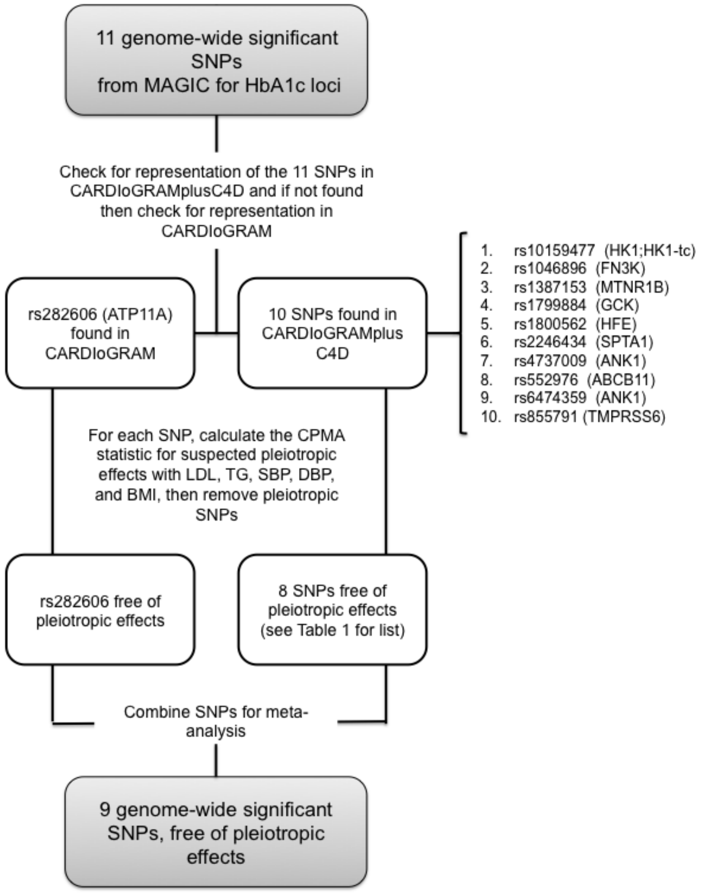

Supplementary Figure 4. Selection and validation of hemoglobin A1c increasing SNPs used as instruments in the Mendelian randomization analysis of the effect of hemoglobin A1c on CHD risk.

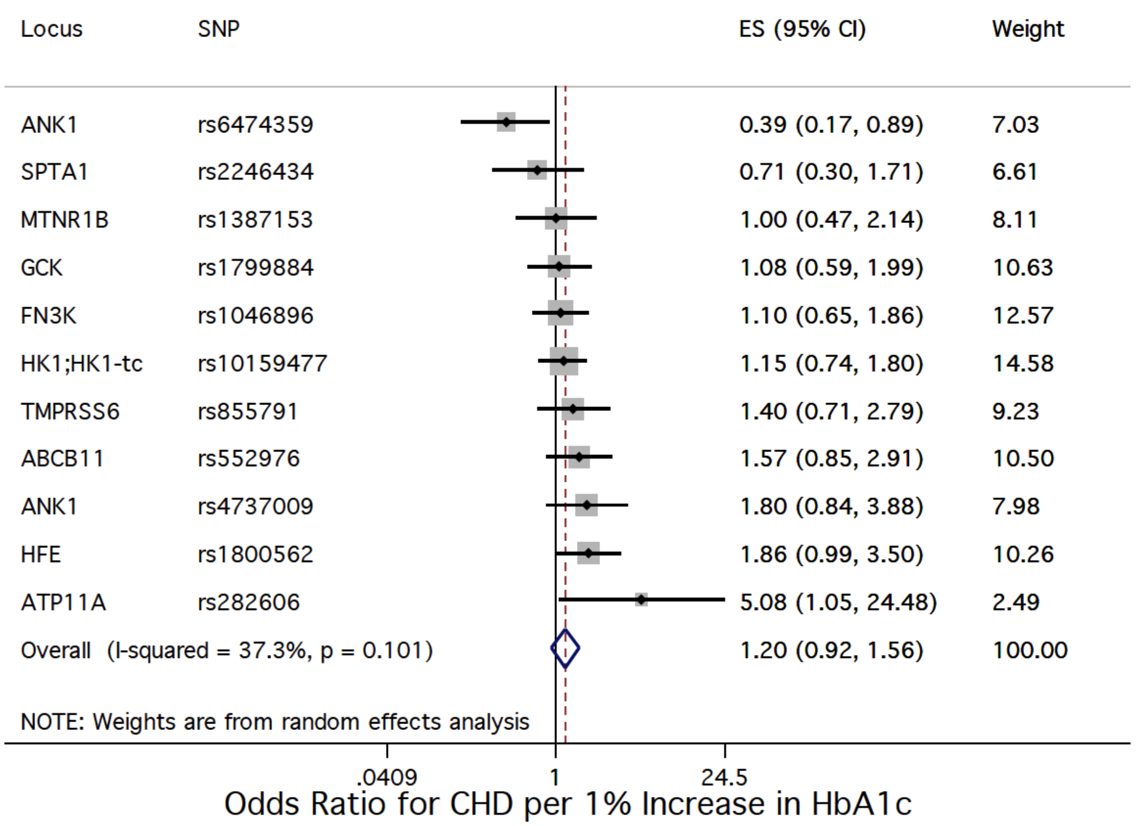

Supplementary Figure 5. The Mendelian randomization estimate of the effect of HbA1c upon CHD using a random-effects model. For each of the 9 non-pleiotropic SNPs (Supplementary Table 5), the Forest plot shows the estimate of the effect of the HbA1c risk allele upon CHD risk, as assessed for each SNP. Also shown for each SNP is the 95% confidence interval (black line segment) of the estimate and the inverse-variance weight (% proportional to the size of the grey square) in the random-effects meta-analysis.

Supplementary Table 1: Random-effects estimate of the typical effect of risk-increasing variants for exposure traits on the exposure.

Exposure	Effect Size (95% CI)	p-value	I^2 (95% CI)
T2D (all significant SNPS, n=37)	1.11 (1.09-1.12)	1.1E-49	88.9 (85.7-91.4)
T2D (excluding pleiotropic SNPS, n=26)	1.10 (1.09-1.11)	1.0E-97	64.9 (46.9-76.9)
Fasting glucose (all significant SNPS, n=33)	0.025 $mmol \cdot L^{-1}$ (0.020-0.031)	1.8E-21	97.3 (96.8-97.8)
Fasting glucose (excluding pleiotropic SNPS, n=24)	0.028 $mmol \cdot L^{-1}$ (0.021-0.035)	9.7E-15	97.9 (97.5-98.3)
Hemoglobin A1c (all significant SNPS, n=11)	0.036 % (0.030-0.043)	1.7E-26	84.8 (74.5-91.0)
Hemoglobin A1c (excluding pleiotropic SNPS, n=9)	0.030 % (0.026-0.035)	1.0E-43	60.3 (17.5-80.9)

Supplementary Table 2A: Association of SNPs from the T2D reference set with confounder traits.

Locus	SNP	LDL	TG	BMI-M	BMI-F	DBP	SBP	CPMA
ADAMTS9	rs6795735	+	+	+	+			
ADCY5	rs11717195	+	+	-	-			
ANK1	rs516946	+	+	+	+			
ANKRD55	rs459193	+	+	+	+			
ARAP1 (CENTD2)	rs1552224	+	+	-	-			
BCAR1	rs7202877	+	+	-	-			
BCL11A	rs243088	+	+	+	+			
CDKAL1	rs7756992	+	+	+	+			
CDKN2A/B	rs10811661	+	+	+	-			
CILP2	rs10401969	+	+	+	+			
DGKB	rs17168486	+	+	+	-			
FTO	rs9936385	+	+	-	-			
GRB14	rs13389219	+	+	+	+			
HHEX/IDE	rs1111875	+	+	-	+			
HMG20A	rs7177055	+	+	-	+			
HMGA2	rs2261181	+	+	-	-			
IGF2BP2	rs4402960	+	+	-	-			
IRS1	rs2943640	+	+	+	+			
JAZF1	rs849135	+	+	+	+			
KCNJ11	rs5215	+	+	+	+			
KCNQ1	rs163184	+	+	+	-			
KLHDC5	rs10842994	+	+	-	+			
MC4R	rs12970134	+	+	+	+			
MTNR1B	rs10830963	+	+	-	-			
PPARG	rs1801282	+	+	-	-			
PRC1	rs12899811	+	+	-	+			
PROX1	rs2075423	+	+	+	+			
SLC30A8	rs3802177	+	+	+	+			
SPRY2	rs1359790	+	+	+	-			
TCF7L2	rs7903146	+	+	-	-			
THADA	rs10203174	+	+	+	+			
TLE1	rs2796441	+	+	+	-			
TSPAN8/LGR5	rs7955901	+	+	-	+			
UBE2E2	rs1496653	+	+	-	-			
WFS1	rs4458523	+	+	-	-			
ZBED3	rs6878122	+	+	+	+			
ZMIZ1	rs12571751	+	+	+	+			

Pleiotropic pathway color legend: white (p > 0.05), light gray (0.001 < p < 0.05), dark gray (0.000001 < p < 0.001), black (p < 0.000001); CPMA: Black represents CPMA p-value < 0.01 for chi-square test with 1 degree of freedom; +/- signs indicate direction of effect. Directions of effect were not available for DBP or SBP.

Supplementary Table 2B: Association of SNPs from the fasting glucose reference set with confounder traits.

Locus	SNP	LDL	TG	BMI-M	BMI-F	DBP	SBP	CPMA
ADCY5	rs11708067	+	+	-	-			
ADRA2A	rs11195502	+	+	+	+			
AMT	rs11715915	+	+	-	-			
ARAP1	rs11603334	+	+	+	+			
CDKAL1	rs9368222	+	+	-	-			
CDKN2B	rs10811661	+	+	+	-			
CRY2	rs11607883	+	+	+	-			
DGKB/TMEM195	rs2191349	+	+	+	-			
DNLZ	rs3829109	+	+	-	-			
FADS1	rs174576	+	+	+	+			
FOXA2	rs6113722	+	+	+	+			
G6PC2	rs560887	+	+	+	+			
GCK	rs2908289	+	+	+	-			
GCKR	rs780094	+	+	-	-			
GIPR	rs2302593	+	+	+	+			
GLIS3	rs10814916	+	+	-	+			
GRB10	rs6943153	+	+	-	+			
IGF2BP2	rs7651090	+	+	+	+			
IKBKAP	rs16913693	+	+	-	+			
KL	rs576674	+	+	+	+			
MADD	rs11039182	+	+	+	+			
MTNR1B	rs10830963	+	+	-	-			
P2RX2	rs10747083	+	+	+	+			
PCSK1	rs4869272	+	+	-	-			
PDX1	rs11619319	+	+	+	+			
PPP1R3B	rs983309	+	+	-	+			
PROX1	rs340874	+	+	-	-			
SLC2A2	rs1280	+	+	+	-			
SLC30A8	rs11558471	+	+	-	-			
TCF7L2	rs7903146	+	+	-	-			
TOP1	rs6072275	+	+	-	-			
VPS13C/C2CD4A/B	rs4502156	+	+	-	-			
WARS	rs3783347	+	+	-	-			

Pleiotropic pathway color legend: white (p > 0.05), light gray (0.001 < p < 0.05), dark gray (0.000001 < p < 0.001), black (p < 0.000001); CPMA: Black represents CPMA p-value < 0.01 for chi-square test with 1 degree of freedom; +/- signs indicate direction of effect. Directions of effect were not available for DBP or SBP.

Supplementary Table 2C: Association of SNPs from the HbA1c reference set with confounder traits.

Locus	SNP	LDL	TG	BMI-M	BMI-F	DBP	SBP	CPMA
ABCB11	rs552976	+	+	+	+			
ANK1	rs4737009	+	+	-	+			
ANK1	rs6474359	+	+	-	+			
ATP11A	rs282606	+	+	-	+			
FN3K	rs1046896	+	+	+	+			
GCK	rs1799884	+	+	+	-			
HFE	rs1800562	+	+	-	-			
HK1;HK1-tc	rs10159477	+	+	-	-			
MTNR1B	rs1387153	+	+	+	+			
SPTA1	rs2246434	+	+	+	+			
TMPRSS6	rs855791	+	+	+	-			

Supplementary Table 3: Fixed-effects model estimates for the effect of T2D and fasting glucose on CHD risk.

Instrument Set	Effect Size (95% CI)	p-value
T2D (all significant SNPs, n=37)	1.10 (1.07-1.13)	4.0E-11
T2D (excluding pleiotropic SNPs, n=26)	1.11 (1.06-1.15)	3.5E-07
Fasting glucose (all significant SNPs, n=33)	1.21 (1.07-1.39)	2.6E-03
Fasting glucose (excluding pleiotropic SNPs, n=24)	1.15 (1.00-1.32)	5.3E-02

Supplementary Table 4. Physiologic clusters of loci identified for T2D. Previously identified clusters were labeled as insulin resistance (IR), hyperglycemic (HG), proinsulin processing (PI), unclassified (UC), and beta-cell dysfunction (BC).

Locus	SNP	Physiologic cluster	Genome-wide significant ($p < 5x10^{-8}$)	Pleotropic effect	Included in study or reason for exclusion
ADCY5	rs11717195	BC	Yes	No	Included
CDKAL1	rs7756992	BC	Yes	No	Included
CDKN2A/B	rs10811661	BC	Yes	No	Included
DGKB	rs17168486	BC	Yes	No	Included
HHEX/IDE	rs1111875	BC	Yes	No	Included
PROX1	rs2075423	BC	Yes	No	Included
SLC30A8	rs3802177	BC	Yes	No	Included
THADA	rs10203174	BC	Yes	No	Included
TCF7L2	rs7903146	BC	Yes	Yes	Suspected pleiotropic effects
MTNR1B	rs10830963	HG	Yes	No	Included
GCK	rs10278336	HG	No	No	Not genome-wide significant
GCKR	rs780094	IR	No	Yes	Not genome-wide significant; suspected pleiotropic effects
KLF14	rs13233731	IR	No	Yes	Not genome-wide significant; suspected pleiotropic effects
IRS1	rs2943640	IR	Yes	Yes	Suspected pleiotropic effects
PPARG	rs1801282	IR	Yes	Yes	Suspected pleiotropic effects
ARAP1 (CENTD2)	rs1552224	PI	Yes	No	Included
BCL11A	rs243088	UC	Yes	No	Included
HMGA2	rs2261181	UC	Yes	No	Included
IGF2BP2	rs4402960	UC	Yes	No	Included
JAZF1	rs849135	UC	Yes	No	Included
KCNQ1	rs163184	UC	Yes	No	Included
PRC1	rs12899811	UC	Yes	No	Included
TSPAN8/ LGR5	rs7955901	UC	Yes	No	Included
WFS1	rs4458523	UC	Yes	No	Included
ZBED3	rs6878122	UC	Yes	No	Included
HNF1B (TCF2)	rs11651052	UC	Yes	No	Not found in outcome
CDC123/ CAMK1D	rs11257655	UC	No	No	Not genome-wide significant
DUSP8	rs2334499	UC	No	No	Not genome-wide significant
HNF1A (TCF1)	rs12427353	UC	No	No	Not genome-wide significant
NOTCH2	rs10923931	UC	No	No	Not genome-wide significant
TLE4	rs17791513	UC	No	No	Not genome-wide significant
ZFAND6	rs11634397	UC	No	No	Not genome-wide significant
TP53INP1	rs7845219	UC	No	Yes	Not genome-wide significant; suspected pleiotropic effects
ADAMTS9	rs6795735	UC	Yes	Yes	Suspected pleiotropic effects
KCNJ11	rs5215	UC	Yes	Yes	Suspected pleiotropic effects

Pleiotropic pathway color legend: white ($p > 0.05$), light gray ($0.001 < p < 0.05$), dark gray ($0.000001 < p < 0.001$), black ($p < 0.000001$); CPMA: Black represents CPMA p-value < 0.01 for chi-square test with 1 degree of freedom; +/- signs indicate direction of effect. Directions of effect were not available for DBP or SBP.

Supplementary Table 5: Characteristics of SNPs considered for use in Mendelian randomization analysis of the effect of HbA1c on CHD risk. Reported p-values are for allelic association of SNPs with each trait.

Locus	SNP	EA	NEA	OR HbA1c	p-value HbA1c	OR CHD	p-value CHD	Pleiotropic Effect
ABCB11	rs552976	G	A	1.03	8.2E-18	1.01	1.5E-01	No
GCK	rs1799884	T	C	1.04	1.5E-20	1.00	8.1E-01	No
MTNR1B	rs1387153	T	C	1.03	4.0E-11	1.00	9.9E-01	No
ANK1	rs4737009	A	G	1.03	6.1E-12	1.02	1.3E-01	No
ANK1	rs6474359	T	C	1.06	1.2E-08	0.94	2.6E-02	No
ATP11A	rs282606	A	G	1.03	1.2E-08	1.05	4.3E-02	No
FN3K	rs1046896	T	C	1.04	1.6E-26	1.00	7.2E-01	No
SPTA1	rs2246434	A	G	1.02	6.0E-09	0.99	4.5E-01	No
TMPRSS6	rs855791	A	G	1.03	2.7E-14	1.01	3.3E-01	No
HFE	rs1800562	G	A	1.07	2.6E-20	1.04	5.3E-02	Yes
HK1;HK1-tc	rs10159477	G	A	1.06	3.2E-25	1.01	5.3E-01	Yes

EA = Effect Allele, NEA = Non-Effect Allele. Pleiotropic Effect: "Yes" indicates that the SNP was associated with at least one confounding trait in the CPMA analysis. See Supplementary Table 1 for a full description of these pleiotropic associations. Note that the OR for CHD is not weighted for the effect of each SNP upon HbA1c. Figures report the OR for CHD weighted by their effect on HbA1c.

Supplementary Table 6: Random-effects model estimates for the effect of hemoglobin A1c on CHD risk. Each p-value is for the MR analyses.

Instrument Set	Effect Size (95% CI)	p-value	I^2 (95% CI)
Hemoglobin A1c (all significant SNPs, n=11)	1.20 (0.92-1.56)	1.7E-01	37.3 (0-69.2)
Hemoglobin A1c (excluding pleiotropic SNPs, n=9)	1.14 (0.82-1.57)	4.3E-01	42.3 (0-73.4)

Supplementary Table 7: Fixed-effects model estimates for the effect of hemoglobin A1c on CHD risk. Each p-value is for the MR analyses.

Instrument Set	Effect Size (95% CI)	p-value
HbA1c (all significant SNPs, n=11)	1.20 (0.98-1.47)	7.2E-02
HbA1c (excluding pleiotropic SNPs, n=9)	1.14 (0.90-1.45)	2.8E-01

Supplementary Note 1. Typical effects risk-increasing genetic variants.

In this section, we present the random-effects estimates of the typical effect of risk-increasing genetic variants for T2D, fasting glucose, and hemoglobin A1c (HbA1c). Because of the high degree of heterogeneity in effect sizes between variants for each of the exposure traits, the random-effects estimates for the typical genetic effect on each exposure trait approaches that of an unweighted average of the individual effect-sizes.

We used a random-effects model to estimate the effect of a typical T2D risk-increasing variant upon T2D risk. Using the full set of genome-wide significant T2D risk-increasing alleles (n=37), we estimated that the typical odds ratio of a T2D risk-increasing variant upon T2D risk to be OR = 1.11 per allele (95%CI: 1.09-1.12); $p = 1.0 \times 10^{-49}$ for the MR analysis; $I^2 = 88.9$ (95%CI: 86-92). Using the subset of non-pleiotropic variants (n=26), we estimated the odds ratio to be OR = 1.10 per allele (95%CI: 1.09-1.11), $P = 1 \times 10^{-97}$ for the MR analysis; $I^2 = 64.9$ (46.8-76.9). (**Supplementary Table 1**, **Supplementary Fig. 1**). As expected, the unweighted average effect for the both the full set of 37 variants and the set of 26 non-pleiotropic variants yielded estimates similar to the random-effects estimates.

We used a random-effects model to estimate the typical effect of fasting glucose-increasing SNPs on fasting glucose in non-diabetic individuals. Using the full set of genome-wide significant SNPs (n=33), we estimated the typical effect size to be 0.025 mmol/L per allele [95%CI: 0.021 - 0.031 mmol/L FG per allele], $P = 1.8 \times 10^{-21}$ for the MR analysis; $I^2 = 97.4$ (95%CI: 96.8-97.8). Using the subset of non-pleiotropic variants (n = 24), we estimated the typical effect size to be 0.028 mmol/L FG per allele [95%CI 0.021-0.035 mmol/L FG per allele]; $P = 9.7 \times 10^{-15}$ for the MR analysis; $I^2 = 97.9$ (97.5-98.3). (**Supplementary Table 1, Supplementary Fig. 2**). As expected, the unweighted average effect for the both the full set of 33 variants and the set of 24 non-pleiotropic variants yielded estimates similar to the random-effects estimates.

Typical effect of HbA1c variants.

We used a random-effects model to estimate the typical effect of HbA1c-increasing SNPs on HbA1c in non-diabetic individuals. Using the full set of genome-wide significant variants (n=11), we estimated the typical effect size to be 0.036% per allele (95%CI: 0.030-0.043% per allele), P $=1.7\times10^{-26}$ for the MR analysis; $I^2 = 84.8$ (95%CI: 74.5-91.0). Using the subset of non-pleiotropic effects (n = 9), we estimated the typical effect size to be 0.030% mmol/L per allele (95%CI 0.026-0.035 mmol/L per allele); $P = 1.0\times10^{-43}$ for the MR analysis; $I^2 = 60.3$ (17.5-80.9). (**Supplementary Table 1, Supplementary Fig. 3**). As expected, the unweighted average effect for the both the full set of 11 variants and the subset of 9 non-pleiotropic variants yielded estimates similar to the random-effects estimates.

Supplementary Note 2. Hemoglobin A1c and CHD risk

We identified 11 genetic variants found to be significantly associated ($P < 5\times10^{-8}$) with HbA1c levels (**Supplementary Table 5, Supplementary Fig. 4)** using data from the MAGIC consortium's most recent GWAS (n = 133,010 non-diabetic individuals).[5] Of these variants, 9 were found to be free of pleiotropy.

Using the full set of genome-wide significant variants (n=11), we carried out a random-effects meta-analysis of the instrumental-variables estimates associated with each SNP. This yielded an estimated effect of OR = 1.2 per 1% increase in HbA1c (95% CI: 0.92-1.56), P = 0.17 for the MR analysis, I^2: 37.3 (0-69.2). Using the non-pleiotropic variants (n=9), we carried out a random-effects meta-analysis of individual instrumental-variables estimates to obtain an MR estimate. This yielded an estimated effect of OR: 1.14 ds per 1% increase in HbA1c (95%CI: 0.82-1.57), p-value = 0.43 for the MR analysis, I^2: 42.3 (95%CI: 0-73.4) (**Supplementary Table 6, Supplementary Fig. 5**). Neither the analysis using the full set of significant variants, nor the analysis using the subset of non-pleiotropic variants produced results that were statistically significant at the 95% confidence level. Fixed-effects models yielded non-significant estimates similar to those of the random-effects models (**Supplementary Table 7**).

Supplementary Note 3. Exposure to T2D in the CARDIoGRAMplusC4D meta-analysis.

The mean age of individuals in the cohorts included in the CARDIoGRAMplusC4D meta-analysis ranged between 45 years and 75.6 years, with a mean of 58.2 years (standard deviation 6.3 years) and an inverse-population-size weighted mean age across cohorts of 59.4 years.[1] For those cohorts for which risk-factor data are publicly available (27 of 37 cohorts), the percentage of patients with T2D ranges from 5% to 42%. The mean age at diagnosis of T2D among white American individuals is 58 years,[2] with a substantial number of patients being diagnosed by the age of 50 years and many with many patients having untreated diabetes for years before diagnosis. Based on these data we feel that, on average, individuals within CARIoGRAMplusC4D were old enough to have developed T2D.

By comparison, in the largest meta-analysis of observational studies to date examining the effect of T2D on cardiovascular disease,[3], encompassing 102 prospective studies and ~ 700 000 individuals, the mean age was 52 yrs. Moreover, a significant finding from this study was that the hazard ratios for coronary heart disease in patients with diabetes were significantly higher at 40–59 years than at 70 years or older.

It is also important to note that exposure to a genetic risk factor for T2D will be present from the time of conception. Consequently, it invariably precedes the disease outcomes that develop in adulthood. Thus, although the design of a Mendelian randomization study is retrospective, the genetic exposure is known, on biological grounds, to precede the outcome.

Both the DIAGRAM and CARDIoGRAMplusC4D sample populations are composed predominantly of individuals of European decent, and hence are largely drawn from the same overall population, justifying our use of separately ascertained effect-sizes. Moreover, a recent trans-ethnic meta-analysis of T2D GWAS studies[4] demonstrates the robustness of the leading DIAGRAM results across most human populations, further supporting our use of effect-sizes determined from the DIAGRAM data-set. Therefore, the available data suggest strongly that the

T2D risk alleles identified in the most recent DIAGRAM publication confer risk of T2D in different cohorts, as well as even different ancestries.

Supplementary References

1. CARDIoGRAMplusC4D Consortium *et al.* Large-scale association analysis identifies new risk loci for coronary artery disease. *Nature Genetics* **45,** 25–33 (2013).
2. Narayan, K. M. V., Boyle, J. P., Thompson, T. J., Sorensen, S. W. & Williamson, D. F. Lifetime Risk for Diabetes Mellitus in the United States. *JAMA* **290,** 1884–1890 (2003).
3. The Emerging Risk Factors Collaboration. Diabetes mellitus, fasting blood glucose concentration, and risk of vascular disease: a collaborative meta-analysis of 102 prospective studies. *The Lancet* **375,** 2215–2222 (2010).
4. DIAbetes Genetics Replication And Meta-analysis (DIAGRAM) Consortium *et al.* Genome-wide trans-ancestry meta-analysis provides insight into the genetic architecture of type 2 diabetes susceptibility. *Nature Genetics* **46,** 234–244 (2014).
5. Scott, R. A. *et al.* Large-scale association analyses identify new loci influencing glycemic traits and provide insight into the underlying biological pathways. *Nature Genetics* **44,** 991–1005 (2012).
